# COL1-Related Disorders: Case Report and Review of Overlapping Syndromes

**DOI:** 10.3389/fgene.2021.640558

**Published:** 2021-05-07

**Authors:** Maria Gnoli, Evelise Brizola, Morena Tremosini, Elena Pedrini, Margherita Maioli, Massimiliano Mosca, Alessandra Bassotti, Paola Castronovo, Cecilia Giunta, Luca Sangiorgi

**Affiliations:** ^1^Department of Rare Skeletal Disorders, IRCCS Istituto Ortopedico Rizzoli, Bologna, Italy; ^2^Orthopedic and Traumatologic Clinic, IRCCS Istituto Ortopedico Rizzoli, Bologna, Italy; ^3^Regional Center of Ehlers-Danlos Syndrome, Fondazione IRCCS Ca' Granda–Ospedale Maggiore Policlinico, Milan, Italy; ^4^Occupational Health Unit, Fondazione IRCCS Ca' Granda–Ospedale Maggiore Policlinico, Milan, Italy; ^5^Medical Genetics Laboratory, Fondazione IRCCS Ca' Granda–Ospedale Maggiore Policlinico, Milan, Italy; ^6^Connective Tissue Unit, Division of Metabolism and Children's Research Centre, University Children's Hospital, Zurich, Switzerland

**Keywords:** COL1, collagen, collagen type I, overlap, clinical signs, musculoskeletal diseases, rare diseases

## Abstract

Collagen type I mutations are related to wide phenotypic expressions frequently causing an overlap of clinical manifestations, in particular between Osteogenesis Imperfecta (OI) and Ehlers-Danlos syndrome (EDS). Both disorders present inter- and intra-familial clinical variability and several clinical signs are present in both diseases. Recently, after the observation that some individuals first ascertained by a suspicion of EDS resulted then carriers of pathogenic variants of genes known to primarily cause OI, some authors proposed the term “COL1-related overlap disorder” to describe these cases. In this paper, we report clinical, molecular, and biochemical information about an individual with a diagnosis of EDS with severe joint hypermobility who carries a pathogenic heterozygous variant in *COL1A2* gene, and a benign variant in *COL1A1* gene. The pathogenic variant, commonly ascribed to OI, as well as the benign variant, has been inherited from the individual's mother, who presented only mild signs of OI and the diagnosis of OI was confirmed only after molecular testing. In addition, we reviewed the literature of similar cases of overlapping syndromes caused by COL1 gene mutations. The reported case and the literature review suggest that the COL1-related overlap disorders (OI, EDS and overlapping syndromes) represent a continuum of clinical phenotypes related to collagen type I mutations. The spectrum of COL1-related clinical manifestations, the pathophysiology and the underlying molecular mechanisms support the adoption of the updated proposed term “COL1-related overlap disorder” to describe the overlapping syndromes.

## Introduction

*COL1A1* and *COL1A2* pathogenic variants have been related to several connective tissue disorders (Table 1), including Osteogenesis Imperfecta (OI) and Ehlers-Danlos syndrome (EDS)^1^^,^^2^. Both are heterogeneous groups of disorders sharing several clinical features, as joint hypermobility, osteoarticular abnormalities, and chronic pain.

**Table 1:** Main diseases related to *COL1A1* and *COL1A2* genes.

**Disorder**	**Phenotype MIM number**	**Gene related**	**Severity**	**Inheritance**	**Age of onset**	**Main clinical signs**	**Other unique signs**
Osteogenesis Imperfecta, type I	166200	*COL1A1* *COL1A2*	Mild	AD	All life spam	Blue sclerae, mild short to normal stature, normal teeth (in most patients), thin skin, onset of fracture usually when child begins to walk, fractures, mild joint hypermobility, hearing loss, mild low mineral bone density, wormian bones	Mitral valve prolapse
Osteogenesis Imperfecta, type II	166210	*COL1A1* *COL1A2*	Lethal	AD	Prenatal/ perinatal	Blue sclerae, severe short stature, short limb dwarfism, severe bone fractures, severe bowing of long bones, platyspondyly, spine deformities, severe low mineral bone density, multiple fractures present at birth, thin skin, wormian bones	Low birth weight, hypotonia, congestive heart failure, pulmonary insufficiency, beaded ribs, large fontanelles, premature birth
Osteogenesis Imperfecta, type III	259420	*COL1A1* *COL1A2*	Severe	AD	Prenatal/ perinatal/lethal form	Normal sclerae, severe short stature, severe bone fractures and refractures, short limb dwarfism, dentinogenesis imperfecta, hearing loss, thin gracile ribs, severe low mineral bone density, long bone deformities, platyspondyly, spine deformities, multiple fractures present at birth, wormian bones	Popcorn calcification, pulmonary hypertension, basilar impression, pseudarthrosis, large anterior fontanelle, delayed gross motor development
Osteogenesis Imperfecta, type IV	166220	*COL1A1* *COL1A2*	Moderate	AD	Perinatal/ infantile	Normal-grayish sclerae, moderate short stature, dentinogenesis imperfecta, otosclerosis, sensorial hearing loss, mild-moderate skeletal deformity, multiple fractures, long bone deformities, spine deformities, wormian bones	
Caffey disease	114000	*COL1A1*	Mild to severe	AD	Prenatal/ infantile	Cortical hyperostosis, swelling of soft tissues around affected bones, pain, fractures, mild long bone bowing	Episode of massive subperiosteal new bone formation accompanied by systemic fever and pain
Ehlers-Danlos Syndrome, Arthrochalasia type, 1 (EDS type VIIa)	130060	*COL1A1*	Mild to moderate	AD	Perinatal	Mild to moderate short stature, severe joint hypermobility, recurrent severe joint dislocations and subluxations, hyperextensible skin, atrophic scars, spine deformities, low bone mineral density	Congenital hips' dislocation at birth, premature osteoarthritis
Ehlers-Danlos Syndrome, Arthrochalasia type, 2 (EDS type VIIb)	617821	*COL1A2*	Moderate to severe	AD	Perinatal	Mild to moderate short stature, severe joint hypermobility, recurrent severe joint dislocations and subluxations, hyperextensible skin, atrophic scars, spine deformities, fractures, low bone mineral density, wormian bones	Congenital hip dislocation, hypotonia, delayed gross motor development, inguinal and umbilical hernia acrogeria (rare), salt and pepper' stippling of calvarium (rare)
Ehlers-Danlos Syndrome, cardiac valvular type	225320	*COL1A2*	Moderate to severe	AR	Infantile–adulthood	Skin hyperextensibility, atrophic scars, thin skin, moderate joint hypermobility, cardiac defects (mitral valve prolapse, mitral regurgitation, mitral valve insufficiency, aortic insufficiency), pectus excavatum	Inguinal hernia, delayed wound healing, muscle and tendon tears
{Osteoporosis, postmenopausal}	120160	*COL1A2*		AD	Adulthood	Severe low bone mass, vertebral fractures, osteoporotic fracture	
{Bone mineral density variation QTL, osteoporosis}	166710	*COL1A1*		AD	Adulthood	Low bone mineral density, low-trauma fractures	

OI (MIM numbers#166200, #166210, #259420, #166220) is a rare connective tissue disorder, characterized mainly by bone fragility and by other signs of connective tissue involvement such as joint hypermobility and heart defects (Forlino and Marini, 2016; Maioli et al., 2019). The *COL1A1* and *COL1A2* genes encode for the α chains of collagen type 1; more than 90% of OI individuals have disease causing variants in these genes (Van Dijk and Sillence, 2014). The most common *COL1A1* or *COL1A2* sequence abnormalities detected in OI are point mutations that affect a glycine residue within the helical domain (Rauch and Glorieux, 2004; Marini et al., 2007).

EDS identifies a group of disorders that affect the connective tissues, including skin, bones, blood vessels, and many other organs and tissues. The main clinical features are joint hypermobility, skin hyperextensibility, and tissue fragility (Malfait et al., 2017). The updated classification of EDS linked collagen type I pathogenic variants include: arthrochalasia EDS–aEDS (associated to *COL1A1* and *COL1A2* mutations); cardiac-valvular–cvEDS (related to biallelic pathogenic *COL1A2* variants); vascular EDS–vEDS (rarely associated to specific *COL1A1* arginine to cysteine substitutions); and classic EDS–cEDS (rarely related to *COL1A1*) (Malfait et al., 2017).

Thus, mutations in the *COL1A1* gene have been found to cause several forms of EDS, moreover, it has been demonstrated that variants in particular regions are related to an OI/EDS overlapping phenotype (Cabral et al., 2007; Malfait et al., 2013). Moreover, mosaic mutation for a lethal OI variant has been described to cause a mild phenotype, with OI/EDS overlapping clinical manifestations (Symoens et al., 2017). *COL1A1* gene mutations have been rarely found in classical EDS (cEDS), whereas *COL1A2* pathogenic variants have never been associated to cEDS (Malfait et al., 2007).

Recently, some authors proposed the term “COL1-related overlap disorder” to describe individuals with clinical suspect of EDS and pathogenic variants in genes known to primarily cause OI (Morlino et al., 2020). The authors described 21 affected individuals with multiple soft connective tissue features, five with heterogeneous variant in *COL1A1* and eight in *COL1A2*. These findings suggested a wider spectrum of clinical manifestations of collagen type I pathogenic variants including intermediate or mixed phenotype between EDS and OI.

Herein we report a family with a benign variant in *COL1A1* and a severe OI causative mutation in *COL1A2* that is related to an EDS phenotype in the proband and to mild OI clinical signs in the proband's mother. Furthermore, we present a review of the literature for similar cases of overlapping syndromes caused by COL1 gene pathogenic variants.

## Case Description

### Clinical Findings

The 30-year-old female proband came to our attention for genetic counseling related to a previous clinical diagnosis of EDS received at 23-year-old. At the age of 19 years she began to undergo clinical evaluations for general pain and chronic asthenia: a hereditary connective tissue disorder was suspected.

**Figure 1:**
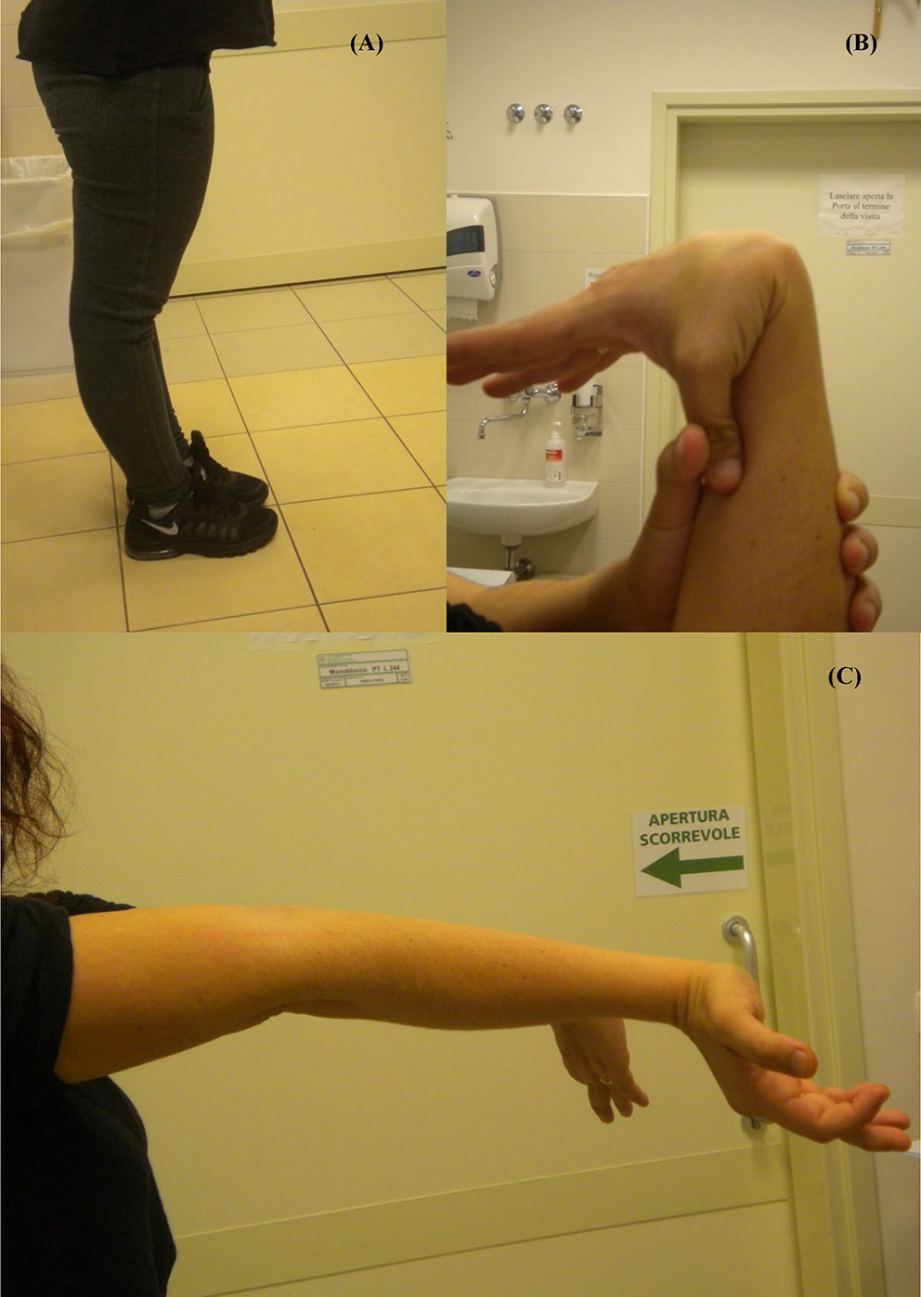
Joint hypermobility: **(A)** Bilateral active hyperextension of the knee; **(B)** Passive apposition of thumb to forearm; **(C)** Active hyperextension of elbow.

She showed generalized joint hypermobility scoring 9/9 in the Beighton score (Figures 1A–C) with recurrent joint dislocations, in particular of shoulders, wrist, and knees. She referred to be prone to bruising and to present a tendency toward prolonged bleeding. Moreover, she was referred with postural hypotension but an autonomic dysfunction was excluded. She had no blue sclera, dental abnormalities or visual defects. An audiological examination revealed a mild hearing loss at high frequencies. Due to recurrent headache, at the age of 26, she underwent an MRI scan of the brain that did not identify any pathological findings.

Neck vessels ultrasound showed a right carotid artery kinking. She underwent cardiac ultrasound that identified a mild valve insufficiency. At 24-year-old, after surgical removal by laparoscopy of multiple ovarian cysts, previously diagnosed with ultrasounds, she developed an enlarged hypertrophic scar in the site of entry of the laparoscope.

Height was 160 cm (>25°p), she did not present any sign of recent or old bone fractures. At 28-years of age she underwent a dual-energy X-ray absorptiometry (DXA) that revealed an early onset of osteopenia (lumbar and femur neck Z scores −2.1).

Calcium, phosphorus, parathyroid hormone, and alkaline phosphatase levels were in the normal range, while 25-(OH)-vitamin D level was low (value: 15,2 ng/mL; deficiency: <20 ng/mL).

She was born with eutocic delivery, the weight at birth was 4,100 g, length 51 cm. Psychomotor development was regular, no hypotonia or motor delay was diagnosed. She referred having joint hypermobility since childhood, as well as flat feet (clinical report is not available).

After the diagnosis and molecular analysis that identified both investigated *COL1A1* and *COL1A2* variants in the proband, her mother underwent specific evaluations. The proband's mother, aged 55-year-old, had not been referred for any clinical problems; she had a stature below the mean of Italian general population [155 cm (10°p)] (Cacciari et al., 2006), and a DXA scan showed a mild osteopenia (lumbar Z score −1.3/T score −2.3 and femur neck Z score −0.7/T score −1.4). She did not present any sign of general joint hypermobility, blue sclerae, dentinogenesis imperfecta or recurrent fractures. In addition, no cardiac or hearing abnormalities were detected in the specific performed exams.

Regarding family history, it was found that a paternal cousin of the proband underwent clinical investigation that excluded OI or EDS diagnosis. No other family members presented clinical manifestations suggesting OI or EDS or other connective tissue disorders.

### Molecular Findings

At first a diagnosis of EDS was suspected and molecular analysis of a gene panel for collagenopathies was performed. The targeted DNA custom panel, with *COL1A1, COL1A2, COL5A1, COL5A2, COL3A1, ADAMTS2, BGALT7, CHST14, PLOD1, PLOD3*, and *TNXB* genes, was designed with the HaloPlex online design tool (SureDesign, Agilent Technologies) and sequenced on MiSeq platform (Illumina, Inc., San Diego, CA, USA) using a Next Generation Sequencing approach.

Due to the complex structure of the *TNXB* locus, EDS molecular diagnostic workflow should include NGS for the non-homologous *TNXB* sequence, long PCR/Sanger sequencing for the *TNXA/TNXB* homology region and *TNXB* deletion/duplication analysis (Demirdas et al., 2017; Micale et al., 2019). The last two molecular diagnostic steps have not been performed because *TNXB*-related classical-like EDS (*TNXB*-clEDS), the ultrarare type of EDS due to biallelic null variants in *TNXB*, has been excluded by the proband's physical examination that revealed the presence of atrophic scarring.

The analysis failed to find an alteration in *COL5A1* and *COL5A2* genes, but two heterozygous variants in collagen type I genes were identified: a c.133C>G (p.Leu45Val) in exon 2 of *COL1A1* gene and a c.2701G>A (p.Gly901Ser) in exon 42 of *COL1A2* gene.

They were confirmed by Sanger sequencing using BigDye Terminator version 1.1 cycle sequencing kit and the 3130xl Genetic Analyzer (Applied Biosystems, Foster City, CA). Sequence traces were aligned with the GenBank reference sequences of the *COL1A1* cDNA (NM_000088.3) and the *COL1A2* cDNA (NM_000089.3).

The pathogenic nature of the variants was evaluated by a series of *in silico* tools included in Varsome that displays automated variant classification according to the guidelines of the American College of Medical Genetics and Genomics and the Association for Molecular Pathology (ACMG/AMP) (Richards et al., 2015; Kopanos et al., 2019). The first variant is classified as Likely Benign, principally because it has been observed in healthy adults and has a benign computational verdict based on nine benign predictions vs. one pathogenic prediction.

The second one has been reported in the Leiden Open Variation Database (LOVD) and Human Gene Mutation Database (HGMD) as pathogenetic and was related to moderate-severe and lethal OI (two affected individuals, respectively OI type IV and type II) (Nuytinck et al., 1997; Marini et al., 2007; Pyott et al., 2011). Varsome classifies it as a Variant of Unknown Significance (VOUS).

The variants affecting glycine residue in collagen type I are usually damaging and related to the more severe forms of the disease. Both variants in the collagen type I genes were found also in the proband's mother, in whom no evidence of mosaic status was found investigating blood and saliva samples.

To better investigate the effect of the variants the proband underwent a skin biopsy for biochemical screening of collagens.

### Biochemical Findings

Collagen was prepared from cultured dermal fibroblasts of the affected individual by metabolic labeling, with [1,2 ^14^C] proline (Perkin Elmer), followed by digestion with pepsin, separation by 5% SDS–PAGE and visualization by fluorography (Lindert et al., 2018). The biochemical analysis of collagen revealed alterations in the production and secretion of type I collagen, which suggest a clinical diagnosis of OI.

**Figure 2:**
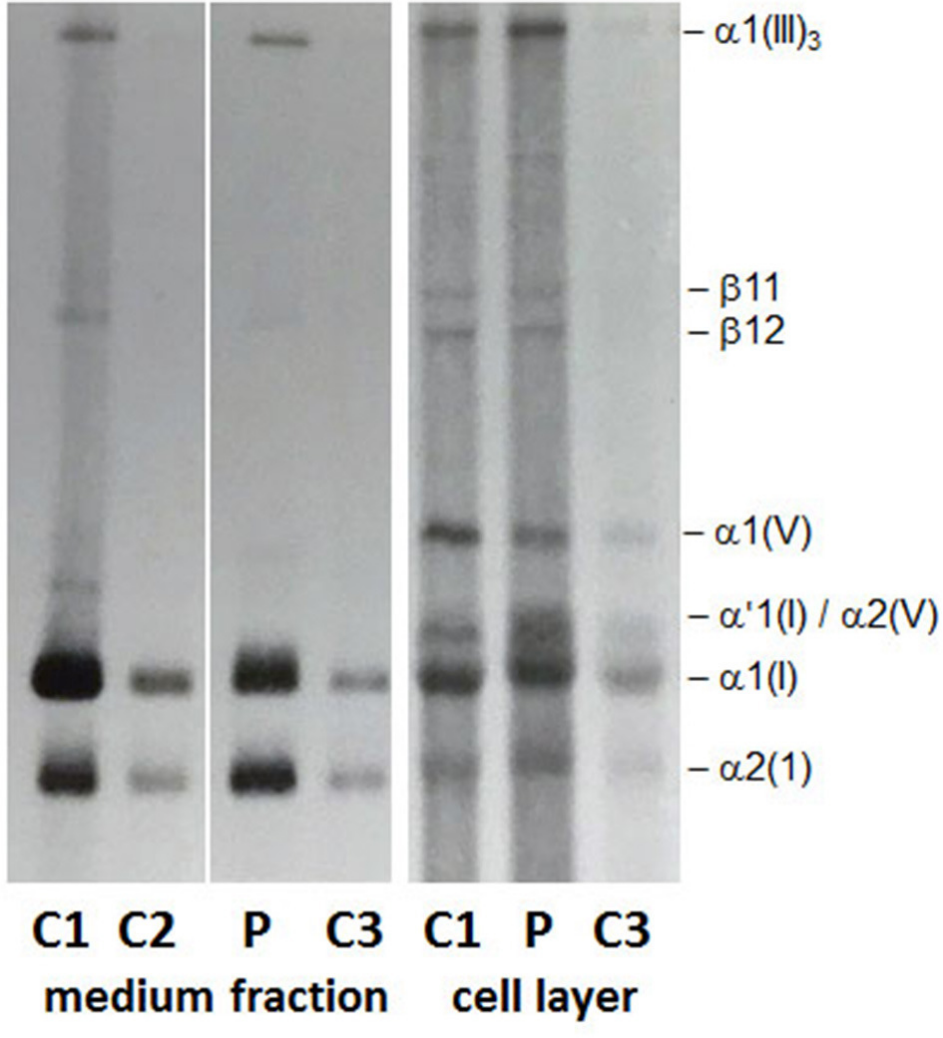
Collagen biochemical analysis: The α1(I) and α2(I) chains in the medium fraction and in the cell layer show a slower migration and a broader appearance on SDS-PAGE. In the cell layer a strong and broad band migrating at the position of a pNα1(I) chain appears to be retained intracellularly.

Figure 2 shows the routine diagnostic SDS-PAGE gel obtained by loading 50 μl of ^14^C proline-labeled collagens extracted from cultured fibroblasts of the patient P and three different controls (C1, C2, and C3). No volume adjustment to show equal band intensity between the different samples has been done. On SDS-PAGE (Figure 2, medium fraction) in the patient fibroblasts (P) both α1(I) and α2(I) chains in the medium fraction as well as in the cell layer presented as overmodified bands with a slower migration and a broader appearance compared to controls (C1, C2, and C3). Furthermore, the cell layer from the patient fibroblasts (P) presented an additional broad α'1(I) band that migrates above the overmodified α1(I) band and appears to be intracellularly retained (α'1(I) in Figure 2, cell layer).

Although, the presence of an undetected variant in either COL1A1 or a collagen chaperon/posttranslational modifying enzyme, that could explain the abnormal findings detected by SDS-PAGE, cannot be completely excluded, we have a tentative alternative hypothesis to explain these abnormal findings. The overmodified α1(I) and α2(I) chains detected on SDS-PAGE in the medium fraction (Figure 2) correspond to the fully processed and secreted triple-helical chains and likely result from the slower formation of the collagen triple helix as a consequence of the COL1A2 p.Gly901Ser mutation. Whereas, the overmodified and intracellularly retained α'1(I) band (Figure 2, cell layer) may represent those overmodified α1(I) chains harboring the *COL1A1* c.133C>G (p.Leu45Val) variant. How this variant in the N-terminal propetide of COL1A1 could interfere with the secretion of the overmodified α1(I) chains should be further investigated and the hypothesis confirmed.

### Review of Similar Cases

A literature review of similar cases performed in PubMed lead to 15 papers published between 1997 and 2020. A summary of the findings and the cases is reported in Table 2.

**Table 2:** Summary of OI/EDS overlap cases reported in the literature.

**Author (Year)**	**Cases**	**Molecular findings**	**Main clinical findings**
Byers et al. (1997)	One individual (child)	*COL1A1* mutations (Intron 5 G>A)	Very marked joint hypermobility, deep blue sclerae, large umbilical hernia
Nicholls et al. (2001)	One individual (adult)	Mutation that substitutes the obligate T+2 of the donor splice site -ctGTAAGT- of *COL1A2* IVS46 with a C	Marked ligamentous laxity and muscle hypotonia at patient's premature (28 weeks' gestation) birth. History of recurrent patellar dislocations, fractures of the skull, pale blue sclerae, clavicle, fingers and toe following minimal traumas
Symoens et al. (2004)	One individual (child)	Skin fibroblasts–A missense mutation (c.3790A>G) in a conserved region of the proa1(I) collagen C-propeptide domain	Fracture of the clavicula and a pneumothorax at birth, motor development delay, blue sclerae, mild hyperelasticity of the skin, mild joint hypermobility mainly at the hands and wrists
Cabral et al. (2005)	Seven individuals (children)	90-residue region at the amino end of the _1(I) collagen chain	Significant short stature, shorter lower extremities, blue sclerae (OI types III or IV), severe large and small joint hypermobility, early progressive scoliosis
Cabral et al. (2007)	Four individuals (One adult and children)	An p.R1066C substitution in one *COL1A1* allele	Low bone mineral density, childhood fractures of long and small bones, light blue sclerae, marked large joint hypermobility
Malfait et al. (2007)	Three individuals (adults)	p.R312C; p.R574C; p.R1093C in *COL1A1* gene	Dissection of medium sized arteries in young adulthood, classic EDS manifestations (one patient), osteopenia (two patients)
Lund et al. (2008)	Three individuals (one adult and two children)	A c.3106C>T substitution in *COL1A1* in exon 44 leading to a p.Arg1036Cys substitution in the a1(I) chain	Joint hypermobility, blue sclerae (one patient), multiple fractures, skin fragility
Malfait et al. (2013)	Seven individuals (one adult and six children)	*COL1A1/COL1A2* mutation within the most N-terminal part of the type I collagen helix	Generalized joint hypermobility and dislocations, skin hyperextensibility and/or translucency, easy bruising, short stature, blue sclerae, low bone mineral density or infrequent fractures
Shi et al. (2015)	Six individuals (one child and five adults)	c.3521C>T (p.A1174V) heterozygous mutation in *COL1A1* gene in a four-generation pedigree	Blue sclerae, skin extensibility, easy bruising, joint dislocations joint hypermobility, fractures, chest deformities, limb deformities, ptosis, flatfoot, congenital cataracts (one patient), dentinogenesis imperfecta (one patient)
Symoens et al. (2017)	One individual (adult)	Mosaic for a small in-frame deletion (c.3150_3158del) in *COL1A1*	Recurrent joint dislocations, mild general joint hypermobility, skin fragility, wound healing delay, tendon rupture, fractures
Lu et al. (2018)	One individual (adult)	Heterozygous *COL1A1* mutation (c.671G>A, p.Gly224Asp) that affected the N-anchor domain of the alpha 1 chain of collagen type I	Short stature, fractures at birth, long bones deformities, motor development delay, grayish-blue sclerae, tooth loss, severe kyphoscoliosis, radial heads dislocation, mild skin hyperextensibility, dislocation of the interphalangeal joints, ligamentous laxity, and generalized joint hypermobility
Lin et al. (2019)	Two individuals (adults)	*COL1A1* (encoding collagen type I α 1 chain) mutation (c.2010delT, p.Gly671Alafs*95)	Multiple long bones fractures, blue sclerae, atrophic scarring, joint hypermobility, prominent ears, easy bruising
Morlino et al. (2020)	21 individuals (five children and 16 adults)	*COL1A1* and *COL1A2* heterozygous variants	Blue sclerae (20 patients), mild long bone bowing (one patient), vertebral fractures (three patients), skin hyperextensibility and fragility, atrophic scars, neonatal hypotonia
Budsamongkol et al. (2019)	One individual (child)	Novel *de novo* missense mutation, c.3296G > A (p.Gly1099Glu) in exon 49 of the *COL1A2* gene	Blue sclerae, bowed legs, multiple fractures, bell shaped chest (OI type III), brachydactyly, dentinogenesis imperfecta, and severe dental developmental disturbance, craniofacial anomalies, highly elastic and fragile skin, generalized joint hypermobility, leg length discrepancy, flat feet
Duong et al. (2020)	One individual (adult)	p.Arg312Cys mutation in *COL1A1*	Generalized joint hypermobility in childhood, with recurrent ankle dislocations, chronic joint pain, multiple fractures, mildly hyperextensible skin, pes planus, severe bilateral hallux valgus, varicose veins, dental fragility and losses

## Discussion

Collagen genes mutations are related to several disorders and to a wide range of clinical and radiological features. Over 1,600 distinct pathogenic variants have been reported in the two genes *COL1A1* and *COL1A2* that encode the two types of polypeptide chain of the type I procollagen proα1(I) and proα2(I) heterotrimer^3^.

Clinical expression variability is a common trait of OI and EDS. Both disorders are collagenopathies and the affected individuals may show many similar features, however the two diseases generally present distinct characteristics. In this article we presented clinical, molecular and biochemical information of an individual with clinical diagnosis of EDS, but a *COL1A2* pathogenic variant usually related to a severe/moderate form of OI and, in addition, the affected individual's mother who had the same *COL1A2* mutation, but only very mild clinical signs of OI.

Since the first case suggesting an overlapping of EDS/OI phenotype was published in 1997 (Byers et al., 1997), other cases have been reported in the literature (Table 2). The use of the term “COL1-related overlap disorder” covers the EDS/OI overlapping phenotypes, other than the common clinical types of EDS or OI already included in the respective classifications (Malfait et al., 2017; Morlino et al., 2020). The mixed phenotype may vary from mild to moderate/severe and major clinical features may include blue sclerae, generalized joint hypermobility, flatfeet with valgus deformity and significantly soft and/or hyperextensible skin. Minor criteria may include hearing loss, short stature, atrophic scars, fractures, joint dislocations, dolichostenomelia, and muscle/ligaments/tendons rupture. Dentinogenesis imperfecta, progressive/severe heart valve disease, congenital fractures, and long bone deformities are considered as exclusion criteria for COL1-related overlap disorder (Morlino et al., 2020).

Similar to other OI/EDS overlap described cases, the affected individual presented joint hypermobility (typical of both OI and EDS), joint dislocations (typical of EDS), skin fragility (typical of EDS), and osteopenia (typical of OI). The proband presented minor signs of vascular involvement such as right carotid artery kinking and a mild valve insufficiency. Cases with vascular involvement, in particular aortic dissection, have been described in OI (Byra et al., 2008; Balasubramanian et al., 2019). A higher rate of vascular events was estimated in overlap OI/EDS cases, in particular, Arginine substitutions in collagen type I have been described in EDS with propensity to arterial rupture (Malfait et al., 2007). Nevertheless, also cases of cEDS without any vascular complication have been reported (Colombi et al., 2017, Duong et al., 2020). The case here reported is comparable to previous reported ones, when initially there is suspicion of EDS on clinical ground but molecular testing for *COL5A1* or *COL5A2* (related to classical EDS) variants are negative and, oddly, variants in *COL1A1* and *COL1A2*, primarily ascribed as causative of OI, are confirmed (Shi et al., 2015; Malfait et al., 2017; Morlino et al., 2020).

The genotype for *COL1A1* and *COL1A2* variants is the same in both proband and proband's mother. The same *COL1A2* mutation has been reported in a case of OI type II and in mosaic state in the clinically not affected parent of the proband (Pyott et al., 2011). However, in this family, no evidence of mosaic status (that might justify the clinical heterogeneity and the very mild expression of the disease in the proband's mother) was found when mutations in DNA extracted from blood sample and saliva was assessed.

The investigation performed to clarify the biochemical outcome of the *COL1A2* and *COL1A1* genetic variants demonstrated alterations in the production and secretion of type I collagen (Figure 2), which could be only partially explained by the known pathogenic effect of the *COL1A2*-mutation. In fact, both α1(I) and α2(I) in the medium and the cell layer presented as overmodified bands, as expected by glycine substitutions within the triple helical domain of collagen that delay collagen triple helix formation (Raghunath et al., 1994). However, the overmodified and slower migrating α'1(I) band that is detected in the cell layer only may represent fully processed and strongly overmodified α1(I)chains that are retained intracellularly and likely degraded. As a consequence, thereof, a reduced amount of overmodified type I collagen molecules is secreted outside of the cell and participate in the formation of mature collagen fibrils and fibers in the extracellular matrix. Based on the hypothesis, yet to be demonstrated, that the p.Leu45Val variant may interfere with the secretion of the strongly overmodified α1(I) chains, we propose to consider it a disease modifier variant with a beneficial effect on the otherwise severe clinical outcome of the *COL1A2* (p.Gly901Ser) variant. This is a speculative interpretation of the unexpected outcome of the biochemical study. However, additional investigations are required to prove this hypothesis, which does not take into account the possibility of the existence of undetected variants in any of the collagen posttranslational modifying enzymes and/or chaperones.

As already highlighted by Morlino et al. (2020), the term OI/EDS overlap may lead to confusion for both non-specialist professionals and affected individuals and, even though it is acceptable on the molecular background, a more specific term such as “COL1-related overlap disorder” could benefit medical management and genetic counseling to for these individuals. These findings corroborate the available literature, suggesting that there is still much more information about the genotype-phenotype correlation in type 1 collagen mutations to be discovered in the future. The case described here reinforces the need of an updated classification for these overlapping EDS/OI phenotypes as “COL1-related overlap disorder” (Morlino et al., 2020).

### Conclusion

We reported the case of an individual with a diagnosis of EDS with severe joint hypermobility who carried a pathogenic heterozygous variant in *COL1A2* gene, and a benign variant in *COL1A1* gene. The pathogenic variant, commonly ascribed to OI as well as the variant with a benign effect on the phenotype, have been inherited from the individual's mother, who showed only very mild signs of OI and the diagnosis of OI could be made only after molecular testing. The literature was reviewed for similar cases of overlapping syndromes caused by COL1 gene mutations. The reported case and the literature review confirmed an extensive clinical expression variability as part of a wider spectrum of clinical phenotypes related to collagen type I pathogenic variants that could justify the adoption of an updated classification as “COL1-related overlap disorder.”

## Data Availability Statement

The pathogenic variant in COL1A2 is reported in the Human Gene Mutation Database – HGMD (http://www.hgmd.cf.ac.uk/ac/index.php). The COL1A1 variant is described on ExAC browser (48277279) (http://exac.broadinstitute.org) and on dbSNP (rs546629502) (https://www.ncbi.nlm.nih.gov/snp/).

## Ethics Statement

Ethical review and approval was not required for the study on human participants in accordance with the local legislation and institutional requirements. The patients/participants provided their written informed consent to participate in this study. Written informed consent was obtained from the individual(s) for the publication of any potentially identifiable images or data included in this article.

## Author Contributions

MG, EB, EP, MMa, MT, MMo, AB, PC, and CG: conception, analysis, collection, and interpretation of data. MG, EB, MMa, PC, CG, and LS: drafting the manuscript. All the authors reviewed and approved the final version of the manuscript.

## Conflict of Interest

The authors declare that the research was conducted in the absence of any commercial or financial relationships that could be construed as a potential conflict of interest. The reviewer FM declared a past co-authorship with one of the authors CG to the handling editor.

## Footnotes

^1^https://www.omim.org/entry/120150?search=COL1A1&highlight=col1a1

^2^https://www.omim.org/entry/120160?search=COL1A2&highlight=col1a2

^3^https://oi.gene.le.ac.uk/
